# PV-IRES-Cre mouse line targets excitatory granule neurons in the cerebellum

**DOI:** 10.1186/s13041-022-00972-1

**Published:** 2022-10-23

**Authors:** Wendy Xueyi Wang, Julia Qiao, Julie L. Lefebvre

**Affiliations:** 1 Program for Neurosciences and Mental Health, Hospital for Sick Children, M5G 0A4 Toronto, Canada; 2 Department of Molecular Genetics, University of Toronto, Toronto, Canada; 3 Institute of Biomedical Engineering, University of Toronto, Toronto, Canada

**Keywords:** Cerebellum, Cre, Interneuron, Granule cell, Reporter, Parvalbumin

## Abstract

Parvalbumin-expressing inhibitory neurons (PV-INs) are critical for the balance and fine-tuning of complex neuronal circuits. Studies of PV-IN biology require tools for their specific labeling, targeting and manipulation. Among these, the Cre/LoxP system is the most popular in mice, with the two commonly used PV-Cre lines cited over 5600 times. Here we report in the mouse cerebellar cortex that PV-Cre activity is not restricted to inhibitory neurons. Imaging of Cre-activated reporters demonstrated recombination in excitatory granule cells. We present evidence that PV-Cre recombination is: (1) spatially regulated and lobule specific; (2) detected in granule cells in the external and internal granule cell layers arising from strong, but transient *Pvalb* expression in progenitors between E13-E15; and (3) delayed in a subset of inhibitory interneurons, asynchronous with PV protein expression. Together, our findings establish the spatio-temporal patterns PV-Cre activation in the mouse cerebellum, raising considerations for conditional targeting of *Pvalb*-expressing inhibitory populations.

## Main text

Parvalbumin (PV), a calcium-binding protein, is widely used for the marking and classification of GABAergic inhibitory interneuron and projection neuron populations throughout the CNS. PV-expressing inhibitory interneurons are critical for maintenance of excitatory-inhibitory balance and circuit outputs [1]. PV is also expressed by populations of GABAergic projection neurons in the cerebellum, basal ganglia and superior colliculus [2, 3]. Together, PV-expressing inhibitory neurons (PV-INs) encompass multiple cell-types with wide-ranging features in morphology, functional activity and molecular signatures [4].

Studies of PV-INs require tools to target and track them during development and at maturity. To this end, development of mouse lines in which Cre recombinase expression is driven under the control of the *Parvalbumin* (*Pvalb)* gene promoter and enhancer elements (hereafter PV-Cre) [5] have enabled studies of diverse aspects of PV-IN biology, including network activity [6], surface receptor function and developmental progression [7, 8]. Despite the broad usage of PV-Cre lines, thorough characterizations of Cre recombination patterns and whether they faithfully target *Parvalbumin*-expressing neurons of interest is still missing. Characterization is particularly important as Cre-dependent recombination is not solely limited to Cre expression at the time of study but can occur with transient or development expression [9]. Moreover, parvalbumin is expressed in select populations of excitatory neurons in the retina, spinal cord, and several brain regions [2, 5].

**Fig. 1:**
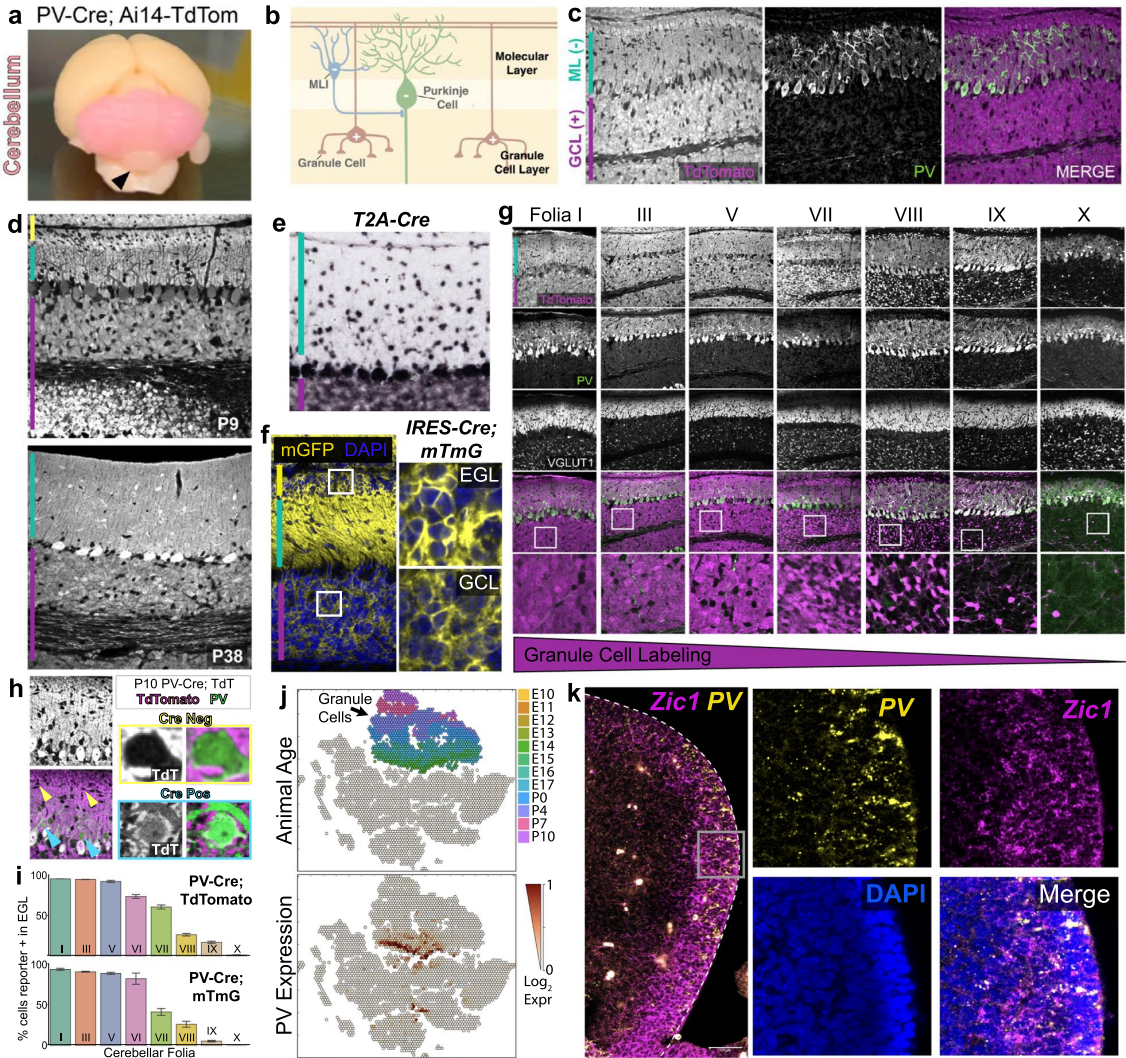
**a** Image of adult (P35) PV-IRES-Cre; Ai14-TdTomato mouse brain. The cerebellum is visibly pink due to high TdTomato expression. Black arrowhead denotes lobule X, only lobule without visible RFP expression. **b** Cartoon outlining the architecture of cerebellar cortex (adapted with modifications from Cerminara et al., Nat. Rev. Neurosci. 2015). Inhibitory neurons (Purkinje projection neurons and molecular layer interneurons, MLIs) are localized to the molecular layer, while excitatory granule cells (GCs) are located in the granule cell layer. **c** Immunostaining of P12 PV-IRES-Cre; Ai14-TdTomato cerebellum. Entire cerebellar cortex is globally TdTomato + with the exception of scattered dark, TdTomato-soma within the molecular layer (ML; containing inhibitory neurons (−); teal bar) and granule cell layer (GCL; containing excitatory neurons ( +); magenta bar). **d** Immunostaining for RFP in PV-IRES-Cre; TdTomato at P9 (top) and P38 (bottom) show TdTomato + granule cells within the GCL (containing mature granule cells; magenta bar) and internal granule cell layer (IGL; containing granule cell progenitors and precursors; yellow bar). **e** TdTomato ISH staining for PV-T2A-Cre; Ai14-TdTomato mouse shows similar positive Cre expression within granule cells (from the Allen Brain Institute). **f** PV-IRES-Cre was crossed to the mTmG mouse line, express membrane-targeted GFP upon Cre recombination (Muzumdar et al., Genesis, 2007). Cerebellar cortex of PV-IRES-Cre; mTmG mouse shows Cre reporter expression in granule cells in the GCL and EGL (insets). High GFP expression in the ML arises from granule cell axons. **g** Lobule specific activation of PV-IRES-Cre in granule cells. Immunostaining for TdTomato, PV, and VGLUT1 are shown for subset of cerebellar folia. VGLUT1 labels GC axon terminals within the molecular layer, and is present across lobules. Zoomed in view of IGL (bottom) show lobule-specific Cre recombination patterns. The majority of granule cells within folia I, III and V are TdTomato + . Few granule cells in folia X are TdTomato + . **h** Immunostaining for PV and TdTomato demonstrate delayed onset of Cre expression in inhibitory interneurons of interest. Migratory MLIs in the upper ML (yellow arrowheads and inset) are TdTomato (magenta) negative despite being positive for PV (green). Maturing MLIs in the lower ML (blue arrowheads and inset) are Cre and PV positive. **i** Quantifications demonstrating lobule-specific nature of PV-Cre recombination. TdTomato + (top) or GFP + (bottom) cells were quantified as a proportion of DAPI + nuclei from the external granule cell layer (EGL) at P9. EGL was chosen as opposed to the internal granule cell layer as it contains almost exclusively granule cell progenitors and precursors, except small numbers of migrating molecular layer interneurons. Error bars represent the standard deviation. **j** UMAP for scRNA-Seq showing *Pvalb* (*PV*) expression within granule cell progenitors between E13–E15. Top panel shows granule cells highlighted, with color codes representing animal age. Bottom panel shows *PV* expression, with high expression colocalizing with the E13–E15 timepoints. **k** Single molecule fluorescence in situ hybridization images for *Zic1* (magenta) and *PV* (yellow) in the E13.5 rhombic lip where granule cell progenitors (GCPs) reside. *Zic1* labels proliferating GCPs. *PV* mRNA is observed in a subset of *Zic1* + GCPs

Here we report that the PV-Cre recombination pattern does not track faithfully with mature PV protein expression. We identified ectopic recombination within cerebellar granule neurons, the most numerous excitatory population in the CNS. To characterize PV-Cre activity, we focused on the widely utilized PV-IRES-Cre mouse line [5], as Cre expression is driven under a knock-in allele from the endogenous PV-promoter (Jax: #017320). We visualized PV-IRES-Cre recombination patterns using the Ai14-Rosa-Cag-TdTomato Cre reporter [10], in which TdTomato expression is induced in Cre-expressing cells. PV-IRES-Cre; Ai14-TdTomato + brains visibly displayed RFP labeling within the cerebellum (Fig. 1a). This high expression pattern was unexpected as PV-expressing neurons encompass less than 1% of the local neuronal population [11].

To determine whether PV-IRES-Cre expression faithfully tracks with PV + populations in the cerebellar cortex, we co-stained cerebellar sections for RFP and Parvalbumin using immunohistochemistry. The cerebellum comprises a stereotyped layered architecture, with PV protein expression restricted to the GABAergic Purkinje projection neurons and molecular layer interneurons (Fig. 1b) [12]. PV proteins are not detected in the granule cell layer (Fig. 1c). By contrast, the TdTomato Cre reporter did not replicate PV protein expression. We found that excitatory glutamatergic granule neurons in the granule cell layer were TdTomato + (Fig. 1c). This pattern was observed in cerebellar tissue analyzed between postnatal day (P)9 and P38 (Fig. 1d). Furthermore, we confirmed that TdTomato expression was present within granule cell precursors within the external granule cell layer at P9, suggesting that PV-IRES-Cre driven recombination occurs during granule cell development (Fig. 1d). A similar recombination pattern is observed for the PV-T2A-Cre line (Fig. 1e; JAX #:012358), and for alternate Cre reporter Rosa-Cag-mTmG (Fig. 1f; JAX # Strain #:007676). Therefore, PV-driven Cre recombination in the granule cell lineage is observed with multiple Cre and reporter lines.

We noted that while the majority of the cerebellar cortex was RFP + , the nodular lobules were spared (Fig. 1a). To determine if this was due to differential, lobule-specific Cre-activation, we further tracked TdTomato expression across all cerebellar folia. While PV-IRES-Cre labeled PV + PC projection and molecular layer interneurons (MLIs) across all cerebellar folia, we identified lobule-specific activation patterns within granule cells. We observed Cre-dependent recombination across all granule neurons in the anterior and central lobules (I–VII), but TdTomato granule cell labeling is sparser in the posterior lobules, with the least cell labeling in lobule X (Fig. 1g). This pattern was observed both within mature granule neurons in the internal granule cell layer, as well as granule cell progenitors and precursors within the external granule cell layer. To confirm that the lack of Cre expression within granule neurons was not due to the absence thereof, we performed co-staining for VGLUT1, a presynaptic marker for granule cell axon terminals in the molecular layer [13]. VGLUT1 staining was high across all cerebellar regions (Fig. 1g). By contrast, within the PV + inhibitory molecular layer interneurons, TdTomato reporter expression was absent or expressed at low levels during the first two postnatal weeks, suggesting a delayed onset of Cre recombination within the cell-types of interest (Fig. 1h). We quantitatively confirmed the lobule-specific variation of PV-Cre activation in presumptive granule cells using both the Ai14-TdTomato and mTmG reporter lines (Fig. 1i). Our findings of TdTomato expression within excitatory neuronal populations cannot be simply assigned to leaky expression or germline recombination of Cre reporter as we did not observe ubiquitous TdTomato expression in other brain regions.

We next investigated whether granule cell progenitors or precursors (GCP) express the *Pvalb* gene during development, leading to Cre expression and reporter activation for the lifetime of the mouse. To this end, we mined published cerebellar single cell RNA-Sequencing (scRNA-Seq) datasets for *Pvalb* expression during embryonic and early postnatal development [14]. Interestingly, we identified strong, but highly transient *Pvalb* mRNA transcripts in GCPs between embryonic days (E)13–E15 Fig. 1j). To confirm this finding, we performed single molecule fluorescence in situ hybridization (smFISH) for *Pvalb* and *Zic*1, a marker for proliferating granule cell progenitors [15]. We observed strong *Pvalb* RNA expression in a subset of *Zic1* + GCPs in the rhombic lip and nascent external granule layer in embryonic mouse brains at E13.5 (Fig. 1k). This finding suggests transient expression of *Pvalb* in the embryonic granule cell lineage and raises potential developmental roles for PV in the excitatory granule cell population.

Taken together, our findings identify transient, embryonic expression of *Parvalbumin* in excitatory granule cells during development. Consequently, PV-Cre recombination in the excitatory granule cell lineage complicates studies requiring conditional manipulations of inhibitory PV + populations in the cerebellum. Alternate strategies or CreER-inducible lines should be considered. We demonstrate the importance of careful spatio-temporal validations of Cre activation patterns when studying targeted neuronal populations.

## Data Availability

All data generated in this study are included in this published article.
